# Mechanical factors contributing to the Venus flytrap’s rate-dependent response to stimuli

**DOI:** 10.1007/s10237-021-01507-8

**Published:** 2021-08-24

**Authors:** Eashan Saikia, Nino F. Läubli, Hannes Vogler, Markus Rüggeberg, Hans J. Herrmann, Ingo Burgert, Jan T. Burri, Bradley J. Nelson, Ueli Grossniklaus, Falk K. Wittel

**Affiliations:** 1 Department of Civil, Environmental and Geomatic Engineering, ETH Zurich, Zurich, 8093 Switzerland; 2 Department of Mechanical and Process Engineering, ETH Zurich, Zurich, 8092 Switzerland; 3 Department of Plant and Microbial Biology and Zurich-Basel Plant Science Center, University of Zurich, Zurich, 8008 Switzerland; 4 Institut für Holztechnologie, 01217 Dresden, Germany; 5 Laboratoire de Physique et Mécanique des Milieux Hétérogènes, École Supérieur de Physique et de Chimie Industrielles de la Ville de Paris, 75005 Paris, France; 6 Swiss Federal Laboratories for Material Science and Technology-EMPA, Cellulose and Wood Materials Laboratory, 8600 Dubendorf, Switzerland; 7 Department of Chemical Engineering and Biotechnology, University of Cambridge, Cambridge, CB3 0AS United Kingdom

**Keywords:** *Dionaea muscipula*, Venus flytrap, Mechanotransduction, Multi-scale modelling, Ion channels, Sensory hair

## Abstract

The sensory hairs of the Venus flytrap (*Dionaea muscipula* Ellis) detect mechanical stimuli imparted by their prey and fire bursts of electrical signals called action potentials (APs). APs are elicited when the hairs are sufficiently stimulated and two consecutive APs can trigger closure of the trap. Earlier experiments have identified thresholds for the relevant stimulus parameters, namely the angular displacement $\theta$ and angular velocity $\omega$. However, these experiments could not trace the deformation of the trigger hair’s sensory cells, which are known to transduce the mechanical stimulus. To understand the kinematics at the cellular level, we investigate the role of two relevant mechanical phenomena: viscoelasticity and intercellular fluid transport using a multi-scale numerical model of the sensory hair. We hypothesize that the combined influence of these two phenomena and $\omega$ contribute to the flytrap’s rate-dependent response to stimuli. In this study, we firstly perform sustained deflection tests on the hair to estimate the viscoelastic material properties of the tissue. Thereafter, through simulations of hair deflection tests at different loading rates, we were able to establish a multi-scale kinematic link between $\omega$ and the cell wall stretch $\delta$. Furthermore, we find that the rate at which $\delta$ evolves during a stimulus is also proportional to $\omega$. This suggests that mechanosensitive ion channels, expected to be stretch-activated and localized in the plasma membrane of the sensory cells, could be additionally sensitive to the rate at which stretch is applied.

## Introduction

The Venus flytrap (*Dionaea muscipula* Ellis) responds to mechanical stimuli imparted by moving insects, which are detected by sensory hairs located on the upper epidermis of the flytrap’s bilobed leaves (Darwin 2014). Typically, there are 3 sensory hairs per lobe and in a constricted region near the base of each hair, there are 30–40 sensory cells (Williams and Mozingo 1971; Haberlandt 1906; Buchen et al. 1983). When insects deflect the hair, the cell walls of the sensory cells are stretched, which causes the mechanosensitive ion channels (MSCs) located in the plasma membrane to open (Sachs 2010; Hedrich 2012; Le Roux et al. 2019). This facilitates ion exchange, leading to an electric potential difference across the membrane called receptor potential (Jacobson 1965). Once the receptor potential reaches a threshold value, an action potential (AP) is fired (Stuhlman and Darden 1950; Böhm et al. 2016; Hodick and Sievers 1988; Burdon-Sanderson 1873). When two such APs occur within 30–40 s, rapid closure of the flytrap is triggered, thereby trapping the insect (Volkov et al. 2007; Di Palma et al. 1961; Brown 1916; Macfarlane 1892).

As reported by (Burri et al. 2020), force sensor-based deflections of the sensory hairs revealed that trap closure depends on the combination of two macro-scale stimulus parameters, namely the angular displacement $\theta$ and the initial angular velocity $\omega$. To assess the influence of a wider range of the two parameters, an electromechanical charge buildup (ECB) model was developed on an analytical framework, based on the concepts of plant electrical memory (Volkov et al. 2009, 2009). By combining test data and ECB model evaluations, we observed that for sufficiently low angular velocities ($\omega < 0.01\,\hbox {rad}\,\hbox {s}^{-1}$), even the highest values of $\theta$ did not result in trap closure. In contrast, for intermediate angular velocities ($0.03\,\,\hbox {rad}\,\hbox {s}^{-1} \le \omega \le 4\,\hbox {rad}\,\hbox {s}^{-1}$), single deflections can trigger trap closure as long as the sensory hair is deflected at least by an appropriate threshold value of $\theta$. Furthermore, no APs were detected during sustained-deflection tests with holding periods of $t=30\,\hbox {s}$. Another recent study reported similar findings, where they detected $\omega$ thresholds equivalent to $0.06\,\hbox {rad}\,\hbox {s}^{-1}$ (Scherzer et al. 2019). Although these observations indicate that the flytrap generates a rate-dependent mechanotransductive response, it is yet unclear how different values of angular velocity $\omega$ at the macro-scale may affect the deformation of sensory cells. Therefore, uncovering the rate-dependent effects at the cellular level can help understanding why a continuous increase (or decrease) in $\theta$ is necessary to elicit APs, while a sustained hair deflection or deflections at sufficiently low $\omega$ are incapable of the same.

In our previous work (Saikia et al. 2021), we developed a multi-scale finite element method (FEM) model of the hair using morphometric data taken from $\mu -$CT images, in order to evaluate the cell wall stretch $\delta$ at threshold values of $\theta$. Since these simulations focused primarily on the influence of morphology on the resulting cell wall stretch, we did not explore the rate-dependent effects at different values of $\omega$. In the present work, we refine our numerical investigations by implementing two relevant mechanical phenomena, namely viscoelasticity of the cell wall and intercellular fluid transport, which may contribute toward the rate-dependent mechanotransductive response of the sensory hair. Thereafter, we simulate force-deflection tests on the FEM hair model for different angular velocities $\omega$. From the simulation outputs, we derive the stretch $\delta$ on the cell walls of the sensory cells and, additionally, trace its evolution during the stimulus. Using this approach, we examine how $\omega$ affects $\delta$, thereby providing insights into the properties of certain MSCs, whose activation may be influenced by the stretch rate.

## Materials and methods

**Fig. 1:**
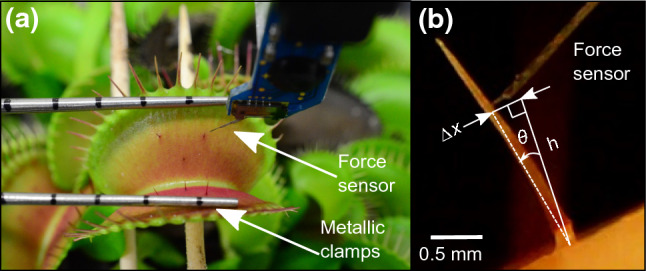
The experimental setup used to characterize the mechanical properties of sensory hairs. **a** The metallic clamps stabilize the lobes to prevent damage to the force sensor in case of trap closure. **b** The force sensor is moved using an xyz positioner and deflects the sensory hair at a height *h* above its base by a horizontal distance $\varDelta x$. This setup is similar to our earlier study (Burri et al. 2020)

In this study, we firstly evaluated the relaxation behavior of the sensory hair *via* sustained deflection tests. Next, we estimated the suitable viscoelastic parameters of the hair’s tissues by calibrating the experimental data with numerical simulations using a FEM model of the sensory hair. Additionally, we implemented an intercellular fluid transport condition between the sensory cells in the model using membrane conductivity data taken from the literature. Finally, the cell wall stretch in the sensory cells was calculated for different stimulus loading rates.

### Sustained deflection tests on sensory hairs

In order to evaluate the relaxation behavior of the sensory hair tissues, we performed sustained deflection tests on 15 hairs, which were collected from 8 different plants, each containing 10–15 pairs of lobes. The Venus flytraps were originally grown from seeds donated by the Botanical Garden Zurich (https://www.bg.uzh.ch) in 2011. The plants were grown in pots having a diameter of 9 cm, and they were kept at 60 % relative humidity and a controlled temperature regime in a greenhouse of the Department of Plant and Microbial Biology of the University of Zurich.

At the beginning of the experiment, we carefully introduced a pair of metallic clamps (see Fig. 1a) to restrict motion of the lobes. This was done to ensure that the force sensor (FT-S1000-LAT, FemtoTools AG) would not get damaged in the event of trap closure. After placing the clamps, the force sensor was placed in contact with the hair at a height *h* (see Fig. 1b) from the base using an xyz positioner (SLC-2475-S, SmarAct). Then, the sensory hairs were horizontally deflected by distances $\varDelta x$: $300\,\upmu \hbox {m}$ (small), $400\,\upmu \hbox {m}$ (medium), and $500\,\upmu \hbox {m}$ (large), respectively, and they were held at these positions for 30 s. The goal behind deflecting the hairs by three different magnitudes of $\varDelta x$ was to detect any significant changes in the relaxation behavior due to possible non-linear viscoelastic effects. Throughout the procedure, the resulting distances and forces were continuously recorded through LabVIEW^TM^. The force sensor was advanced horizontally at a speed of $11\,\hbox {mm s}^{-1}$, as, according to (Burri et al. 2020), no trap closure is initiated at this speed through a single deflection. Note that the hairs of the lobe were manually stimulated after each experiment to verify that the flytraps were in a healthy state and could be triggered successfully.

Two USB microscopes (DigiMicro Profi and DigiMicro, DNT) were used for visual feedback during the manual positioning of the sensor as well as to observe the deflection procedure. From the captured images, we measured the contact height *h* and calculated the angular displacement $\theta =tan^{-1}(\varDelta x/h)$. The equivalent values for $\theta$, corresponding to $\varDelta x$, were classified into three categories: small ($0.1\,\hbox {rad}\le \theta \le {0.15}\,\hbox {rad}$), medium ($0.15\,\hbox {rad}\le \theta \le {0.2}\,\hbox {rad}$), and large ($0.2\,\hbox {rad}\le \theta \le {0.25}\,\hbox {rad}$). Using the force-relaxation data obtained from the deflection tests, we calculated the variation in the reaction moment $M = F\times h$ during the 30-s time period. Thereafter, we evaluated the characteristic decay time of each relaxation curve using a non-linear least squares fit in MATLAB 2019a.

**Fig. 2:**
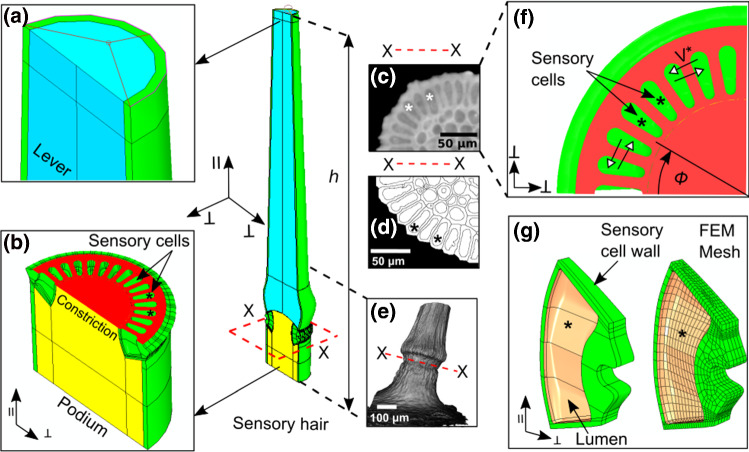
The half-symmetric FEM model of the sensory hair built up to height *h* comprising of different tissue regions. **a** The lever tissue of the hair. **b** Podium and constriction tissues at the bottom. Plane X-X makes a cross-sectional cut (red) through the constriction, wherein the sensory cells (*) are seen. **c** $\mu -$CT image of the hair cross section at X-X. **d** Segmentation of internal cells after post-processing of $\mu -$CT data. **e** 3D rendering of the sensory hair, depicting the podium, constriction, and a section of the lever tissue. **f** Cross-sectional view (X-X) in the FEM model showing the constriction, where sensory cells are embedded. White arrow heads demarcate intercellular fluid transport. **g** 3D geometry of a sensory cell with inner cell wall surface (beige) and cell walls (green). This model is a refinement of the earlier model used for static simulations (Saikia et al. 2021)

### Multi-scale FEM model of the sensory hair

We built a multi-scale numerical model of the sensory hair in the commercial finite element method (FEM) software ABAQUS 2019 to simulate the mechanical stimulus and observe the resulting deformation of the sensory cells. The model comprises of the lever tissue, the podium tissue and, lastly, the constriction tissue, where 16 sensory cells are embedded radially (see Fig. 2a, b). Two additional strategies were adopted for reducing computational costs: Firstly, a half-symmetric geometry of the sensory hair is considered and, secondly, the region of the lever tissue above the contact height $h={1000}\,\upmu \hbox {m}$ was neglected. The morphology of the tissue and the cells in the sensory hair model were obtained from $\mu -$CT scans (see Fig. 2c–e) of three sensory hairs (Saikia et al. 2021). The material properties of the cell wall, the lever, and the podium were estimated previously *via* a parametric study (Saikia et al. 2021), and they are defined by their elastic moduli: $100\,\hbox {MPa}$, 8 MPa, and 16 MPa, and their Poisson’s ratios: 0.3, 0.39, and 0.47, respectively. The lumen of these cells are filled with a compressible fluid, here assumed to be water with a density $\rho = 1000\,\hbox {kg}\,\hbox {m}^{-3}$ and a bulk modulus of 2.1 GPa which exerts a turgor pressure of $P = 0.2\,\hbox {MPa}$. Thereafter, displacements and rotations of the bottom surface of the hair are constrained by setting all nodal degrees of freedom to zero. Additionally, a planar symmetry boundary condition is imposed on the surfaces that lie on the bending plane of the model in order to restrict out-of-plane movements during the deflection. Following the model initialization, we evaluated the suitable viscoelastic material parameters for the cell wall and the hair tissues using the procedure described in Sect. 2.2.1. Note that the hair tissues are modeled as a homogeneous isotropic material for simplicity.

#### Simulation of sustained deflection tests for estimating viscoelastic properties

To estimate the viscoelastic properties of the sensory hair tissues, we performed a parametric study where different stress-relaxation rates were defined for the sensory hair tissues. The relaxation rates were represented by a Prony-series expansion of the shear-relaxation modulus similar to those previously reported by (Yamamoto et al. 1970) for plant cell walls. In our simulations, the hair was deflected up to an angular displacement of 0.15 rad and was held at this position for a total time $t=30\,\hbox {s}$, during which the reaction force *F* at the sensor tip was continuously sampled from the simulation output. Thereafter, the reaction moment $M = F \times h$ was calculated before being normalized by $M_{0}$, which corresponds to *M* at $t=0\,\hbox {s}$. In order to compare the experiments and the simulations, the variation of $M/M_{0}$ over the time *t* was plotted as semi-log curves. Then, the initial linear slopes *k* of the semi-log curves were used to compare their rate of decay. The short-range ($t< 1\,\hbox {s}$) relaxation behavior of the tissues is of special interest because the stimuli imparted by the Venus flytrap’s natural prey are expected to be within this short range.

#### Implementation of fluid transport between sensory cells

A dynamic explicit solver is chosen for capturing the intercellular fluid transport (FT) between sensory cells present in the constriction tissue of the hair (see Fig. 2f). Continuum 3D elements ’C3D8’ are used for discretizing the cell walls (see Fig. 2g), and the overall mass flow rate $m^{*}$ across the cell walls is calculated as:

1 $$\begin{aligned} | m^{*} |= \rho \cdot V^{*} \cdot A, \end{aligned}$$

where $V^{*}$ is the volumetric flow rate per unit area *A* (Smith 2009). This is implemented in the model by defining $V^{*}$ as a linear function of $\varDelta P$, such that $V^{*}= L_{p} \cdot ~| \varDelta P |~$, where $L_{p}$ is the hydraulic conductivity of the membrane (Dumais and Forterre 2012). The two test cases correspond to, firstly, $L_{p}=0\,\hbox {ms}^{-1}\,\hbox {Pa}^{-1}$ (no FT), and secondly, $L_{p}={8e-12}\,\hbox {ms}^{-1}\,\hbox {Pa}^{-1}$ (FT), which is near the upper limit of values for $L_{p}$ reported in the literature (Steudle 1989; Dumais and Forterre 2012).

The mechanical stimulus is imparted on the top surface of the lever (see Fig. 2a) as a horizontal displacement. This approach provides a simple alternative to modeling the force sensor while avoiding inertial effects, which may occur due to initial contact instabilities. To further reduce such inertial effects, the displacement stimulus is defined as a sigmoidal loading function. This way, the hair is deflected up to a maximum value of $\varDelta x = {150}\,{\upmu }\hbox {m}$ (or $\theta ={0.15}\,\hbox {rad}$), after which the hair is retracted back to its original position in the same way. The first set of simulations in this study is aimed at separately understanding the influences of viscoelasticity and fluid transport on sensory cell deformation. Hence, four different cases are considered with the following combinations: (i) elastic cell walls (E), (ii) viscoelastic cell walls (V), (iii) elastic cell walls with fluid transport (E+FT), and (iv) viscoelastic cell walls with fluid transport (V+FT). In all four cases, the evolution of recoverable strain energy (SE) in the sensory cells is sampled as an output during the advance and retreat of the hair.

### Calculation of cell wall stretch at different stimulus loading rates

Membrane stretch is an important mechanical parameter because it is responsible for the opening of the mechanosensitive ion channels (MSCs) (Sachs 2010; Haswell et al. 2011) present in the sensory cells of the trigger hair (Suda et al. 2020; Procko et al. 2021). Therefore, we are interested in the stretch produced on the cell walls (see Fig. 2g) of the sensory cell as well as in quantifying its variation during the deflection simulations. In order to calculate the cell wall stretch, the logarithmic strain values at the FEM mesh nodes, which lie on the inner surface of the cell wall, are monitored. These nodal strains are accessed from the output database by post-processing with the ABAQUS-Python interface. Then, for each element $i\in [1,n]$ on the inner cell wall, a single strain tensor is calculated by averaging the contributions from its associated nodes. Next, a plane-stress condition is imposed on the elements and their in-plane strain components $e_{1}^i,e_{2}^i$ are calculated after transforming the strain tensor into the local coordinate system. Thereafter, the stretch $\lambda ^i$ is computed for a single element *i* using the relation:

2 $$\begin{aligned} \lambda ^i=(1+e_{1}^i)\cdot (1+e_{2}^i). \end{aligned}$$

In this way, $\lambda ^i$ is computed for all the elements on the inner cell wall of all the 16 sensory cells. Following this, we calculated the weighted mean of $\lambda ^i$ with respect to the area fraction, which results in a scalar value corresponding to the cumulative stretch $\delta$. This can be mathematically represented by

3 $$\begin{aligned} \delta = \frac{\sum _{i=1}^{n}(\lambda ^i\cdot A^i)}{\sum _{i=1}^{n}A^{i}}, \end{aligned}$$

where $A^i$ is the area of the element *i*. Using Eqn. 3, we evaluated the variation of $\delta$ for the four combinations of E, V, and FT, as mentioned in Sect. 2.2.2. The next set of simulations explore the combined effect of viscoelastic cell walls and intercellular fluid transport (V+FT) on the evolution of the net cumulative stretch $\varDelta \delta$ for different stimulus loading rates. In these simulations, the deflection amplitude follows the same sigmoidal function as in the case of Sect. 2.2.2 with a maximum angular displacement of $\theta ={0.15}\,\hbox {rad}$. The loading rates are defined by three different angular velocities: $\omega ={0.1}\,\hbox {rad}\, \hbox {s}^{-1}, {0.5}\,\hbox {rad}\, \hbox {s}^{-1}$, and ${1}\,\hbox {rad}\, \hbox {s}^{-1}$. Lastly, we calculated $\psi _{\omega }$ from the slope of $\varDelta \delta$ *vs.* $\theta$ in the linear region $\theta \in [0.06 ,0.12]$ of the sigmoidal deflection function.

## Results

**Fig. 3:**
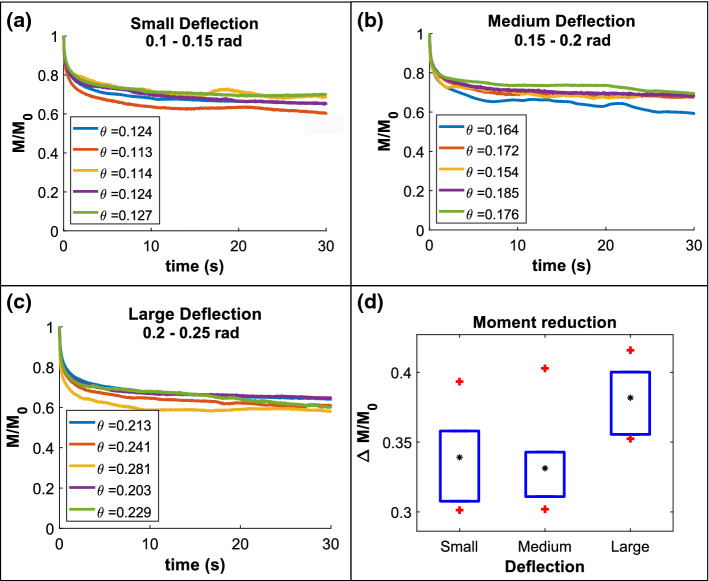
Decay in normalized reaction moment ($M/M_{0}$) measured during sustained deflection experiments at **a** small, **b** medium, and **c** large deflections. The hair is held at its maximum angular displacement for 30 s. **d** Boxplot showing the reduction in the normalized reaction moment ($\varDelta M/M_{0}$) for small, medium, and large deflections. Black stars correspond to the mean value and red plus signs denote the most extreme values

**Fig. 4:**
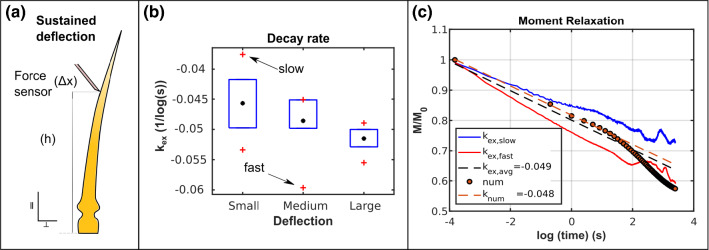
**a** Deflection of the hair up to a horizontal distance $\varDelta x$ by a force sensor in contact at a height *h*. **b** Boxplot of the initial linear slope ($k_{ex}$) of the semi-log plots of $M/ M_{0}$ *vs.* log(*t*) obtained from sustained deflection experiments. The mean values are depicted by black circles. **c** $M/ M_{0}$ *vs.* log(*t*) curves for minimum (fast, red) and maximum (slow, blue) values of the slope $k_{ex}$. The black dashed line denotes the mean value $k_{ex,avg}$ of all the experimentally measured decay rates, while the orange dashed line corresponds to the numerical decay rate $k_{num}$

### Decay in reaction moments during sustained-deflection tests

We performed sustained deflection tests on a total of 15 sensory hairs with small (*n* = 5), medium (*n* = 5), and large (*n* = 5) angular displacements $\theta$ using the procedure described in Sect. 2.1. Thereafter, the reaction moments *M* were calculated from the reaction force data, which was continuously measured at the sensor tip during the sustained deflection period of 30 s. The decay in the value of the normalized moment $M/M_{0}$ is plotted in Fig. 3a–c for the three categories of deflection angles: small, medium, and large.

**Table 1:** Prony-series constants $\tau _{1}$ and $\tau _{2}$ (mean ± std. dev.) are reported for moment relaxation curves at small, medium, and large deflections of the sensory hair

Hair deflection	$\tau _{1}$ (s)	$\tau _{2}$ (s)
Small; $\theta =$ [0.1–0.15] rad	0.24 ± 0.09	6.77 ± 2.37
Medium; $\theta =$ [0.15–0.2] rad	0.32 ± 0.07	11.50 ± 5.16
Large; $\theta =$ [0.2–0.25] rad	0.25 ± 0.07	9.36 ± 3.56

By fitting a two-term Prony-series expansion to the variation of $M/M_{0}$ over time *t*, we found the short-term and long-term decay constants $\tau _{1}$ and $\tau _{2}$, respectively (see Table 1). In particular, we are interested in the short-term relaxation behavior of the sensory hair, which is then used to compare the tests with our numerical simulations in Sect. 3.2. The reason behind our focus on only the short-term relaxation behavior is that a single touch stimulus is capable of eliciting trap closure within 1–2 s of stimulation time, as revealed by previous deflection experiments (Burri et al. 2020).

Furthermore, at the end of 30 s of sustained deflections, the relaxation of the sensory hair tissues results in a reduced final value of the reaction moment, which is computed as $\varDelta M = M_{0}-M_{30}$. The normalized moment reduction $\varDelta M/ M_{0}$ for each of the three categories is depicted by a boxplot (see Fig. 3d), with each box comprising five tests on independent samples. The mean values of $\varDelta M/ M_{0}$ are marked with black stars on the boxplots and were derived as $0.339\pm 0.033$, $0.331\pm 0.036$, and $0.382\pm 0.024$ for small, medium, and large deflections, respectively. Since the mean values of $\varDelta M/ M_{0}$ for the three deflection categories did not bear significant differences, we assumed a linear viscoelastic behavior for the sensory hair tissues.

### Estimation of viscoelastic material parameters of the sensory hair tissues in the FEM model

In order to ensure that our numerical model is able to capture the short-term hair relaxation of the sensory hair, we calibrated the model by comparing the sustained deflection simulation outputs with the relaxation test data, which allowed us to estimate suitable parameters for the viscoelastic material properties of the sensory hair tissues. Figure 4a shows a plot of the experimental sustained deflection tests that has also been reproduced as described in Sect.  2.2.1 using the FEM hair model.

We quantified the short-term relaxation behavior of the sensory hair by calculating the decay rates $k_{ex}$ measured from the initial linear slopes of the semi-log decay plots of $M/ M_{0}$ over time *t* (shown in Fig. 4c). Boxplots of the decay rates are presented in Fig. 4b, where the average value of the decay rates for small, medium, and large deflections are demarcated by black circles. Among all 15 sustained deflection experiments, the fastest and the slowest decay rates are $k_{ex, fast} = -0.0596$ (1/log(s)) and $k_{ex, slow} = -0.0376$ (1/log(s)), respectively, which are represented by the blue and the red semi-log plots in Fig. 4c. Furthermore, the average value of all the decay rates is depicted in Fig. 4c by the black dashed line corresponding to $k_{ex, avg} = -0.049\pm 0.0052$ (1/log(s)). In the same figure, the semi-log plot of $M/ M_{0}$ over *t* for the numerical simulations of sustained deflection tests is marked by orange circles. The two-term Prony-series decay constants used as input parameters for the model are $\tau _{1}={0.74}\,\hbox {s}$ and $\tau _{2}={1.06}\,\hbox {s}$. From the semi-log plot, the initial linear slope for the numerical output (orange dashed lines) is calculated as $k_{num} = -0.048$ (1/log(s)).

The measurements made by the force sensor are limited to macro-scale parameters, namely the angular displacement $\theta$ and reaction force *F* at the sensor tip, which does not inform us about changes at the cellular level. Therefore, in the next step (Sect. 3.3), we examine the effect of hair deflection on the sensory cells by simulating a single deflection stimulus.

### Influence of intercellular fluid transport and viscoelastic material behavior

**Fig. 5:**
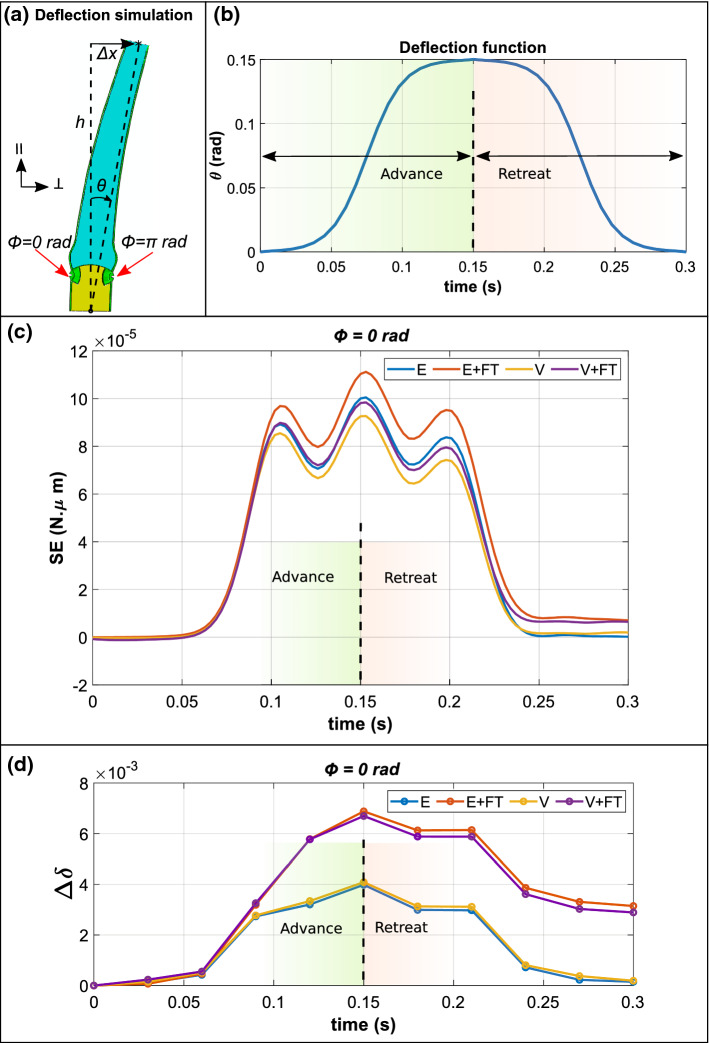
**a** ABAQUS viewport image depicting the simulation of single deflection stimulus on the hair model by imparting a horizontal distance $\varDelta x$, leading to an angular displacement $\theta$. **b** Sigmoidal deflection function of the angular displacement $\theta$ during the single deflection stimulus over a total time of 0.3 s. **c** Variation of recoverable strain energy (SE) in the cell under tension, located at $\phi ={0}\,\hbox {rad}$ during the deflection. The four cases considered are E (blue), E+FT (orange), V (yellow), and V+FT (purple). **d** Variation of net cumulative stretch $\varDelta \delta$ during the advance and retreat of the hair, shown for the four cases mentioned above

Once the viscoelastic material properties are determined in Sect. 3.2, a fluid exchange condition was implemented (see Sect. 2.2.2), followed by simulations of the force-deflection experiments using the FEM model. Hair deflection can be divided into two phases, *i.e.*, the advance phase followed by the retreat phase. Firstly, the sensory hair is advanced by using a sigmoidal function of $\theta$ *vs.* *t* (see Fig. 5b) for a duration of $t={0.15}\,\hbox {s}$ up to a maximum angular displacement of $\theta ={0.15}\,\hbox {rad}$. The retreat phase follows immediately for the next 0.15 s, at the end of which the sensory hair is back at its initial position.

**Fig. 6:**
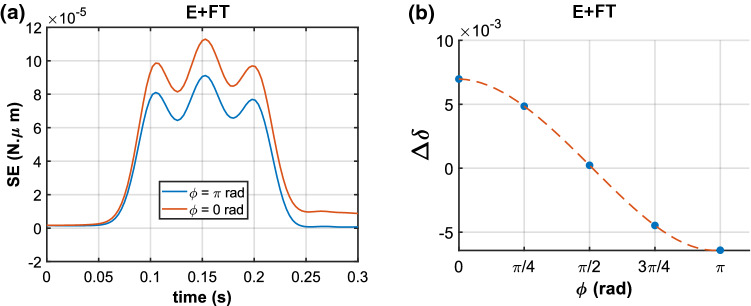
Effect of stimulus on different sensory cells of the E+FT model. **a** Variation of strain energy (SE), shown for the two sensory cells located on the bending plane at $\phi ={0}\,\hbox {rad}$ (orange, under tension) and $\phi ={\pi }\,\hbox {rad}$ (blue, under compression). **b** Net cumulative stretch rate $\varDelta \delta$ at maximum angular displacement (time point $t={0.15}\,\hbox {s}$) in five different sensory cells, each located at intervals of $\phi =\pi /4$ rad

During the advance phase of the hair deflection (see Fig. 5a), the sensory cell located at $\phi ={0}\,\hbox {rad}$ is under maximum tension and, hence, it is likely that its cell walls also experience the highest stretch. Consequently, we focus our subsequent observations on the cell at $\phi ={0}\,\hbox {rad}$. As the hair is deflected, the cell walls of the sensory cells deform and the recoverable strain energy (SE) stored in the cell walls increases as the hair advances up to $\theta ={0.15}\,\hbox {rad}$. In Fig. 5c, the variation (smoothed by spline) of SE for the sensory cell located at $\phi ={0}\,\hbox {rad}$ is shown as a function of angular displacement $\theta$. As the sensory hair retreats, the value of SE reduces as the hair resumes its initial orientation at $\theta ={0}\,\hbox {rad}$.

We simulated four cases with different properties of the sensory cells which include: linear elastic cell walls without fluid transport (E), linear elastic cell walls with fluid transport (E+FT), viscoelastic cell walls without fluid transport (V), and, lastly, viscoelastic cell walls with fluid transport (V+FT). Upon comparing the SE values derived from the four cases in Fig. 5c, E+FT (blue) attained the highest SE value $\sim 11\times 10^{-5}\,\hbox {N}\cdot \upmu \hbox {m}$ at a maximum deflection of $\theta ={0.15}\,\hbox {rad}$. At the same time, for case V, consisting of viscoelastic cell walls without fluid transport (yellow), the SE has the lowest value of $\sim 9 \times 10^{-5}\,\hbox {N}\cdot \upmu \hbox {m}$ at a deflection angle of $\theta ={0.15}\,\hbox {rad}$. These four cases can, therefore, be arranged in the ascending order of their SE values at maximum deflection as V < V+FT < E < E+FT. In all the cases, it can be seen that fluid transport leads to higher SE for both elastic and viscoelastic cell walls.

Figure 5d shows the variation of net cumulative stretch $\varDelta \delta$ for the four combinations during the advance and retreat of the hair. The influence of FT on the stretch is visibly larger than when FT is deactivated. When the hair is at its maximum deflection, marked by $t=0.15\,\hbox {s}$, the values of $\varDelta \delta$ for both E+FT (orange) and V+FT (purple) are 1.72 and 1.63 times higher than their counterparts E and V, respectively. At the same time, $\varDelta \delta$ values for E+FT is higher than V+FT by a relatively small factor of 1.03. Furthermore, the effect of FT leads to consistently higher values of $\varDelta \delta$ during the hair’s retreat.

During the single deflection stimulus, the variation of SE in the 16 sensory cells differs in magnitude, with limiting values at $\phi ={0}\,\hbox {rad}$ (max) and $\phi ={\pi }\,\hbox {rad}$ (min) (see Fig. 6a). Unlike the cell at $\phi ={0}\,\hbox {rad}$, which has a residual SE of nearly $1\times 10^{-5}\,\hbox {N}\cdot \upmu \hbox {m}$ at the end of the retreat, there is no residual SE at $\phi ={\pi }\,\hbox {rad}$. This difference arises because there is no additional fluid in the cell at $\phi ={\pi }\,\hbox {rad}$ during the retreat, owing to the initial fluid efflux when the cell is compressed. The residual SE in the remaining 14 cells demonstrated a progressive increase from $\phi ={\pi }\,\hbox {rad}$ to $\phi ={0}\,\hbox {rad}$.

The compression of the cells located at $\phi \ge {\pi /2}\,\hbox {rad}$ during the advance of the hair, results in negative values of their net cumulative stretch $\varDelta \delta$. In Fig. 6b, it can be seen that at maximum angular displacement of $\theta ={0.15}\,\hbox {rad}$ ($t={0.15}\,\hbox {s}$), the cell at $\phi ={\pi }\,\hbox {rad}$ attains a $\varDelta \delta$ value of $-6.41 \times 10^{-3}$, whereas $\varDelta \delta$ for the cell at $\phi ={0}\,\hbox {rad}$ is $6.97 \times 10^{-3}$. This indicates that the contraction of the cell walls of compressed cells is nearly equal in magnitude, yet opposite in direction, to the stretch in the cells under tension. Note that, $\varDelta \delta$ at $\phi ={\pi /2}\,\hbox {rad}$ is $\sim 0$, because the cell is simultaneously under tension and compression.

### Evolution of sensory cell wall stretch due to different stimuli loading rates

**Fig. 7:**
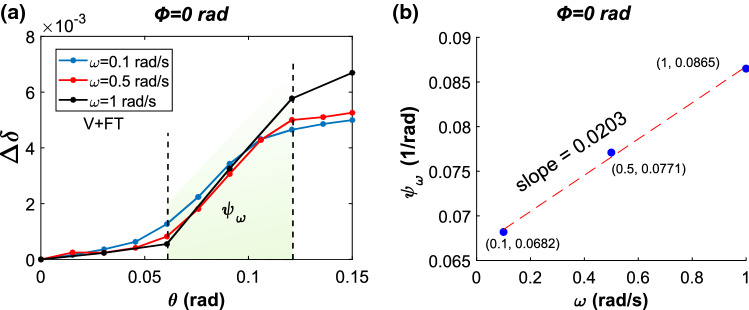
Simulation outputs are used to compute **a** net cumulative cell wall stretch $\varDelta \delta$ for V+FT in the sensory cell under the highest tension, located at $\phi ={0}\,\hbox {rad}$, due to hair deflection up to $\theta ={0.15}\,\hbox {rad}$ for different $\omega$. Note that the black curve corresponds to the purple curve in Fig. 5d for $\omega = {1}\,\hbox {rad s}$. **b** Stretch rate $\psi _{\omega }$ for three different values of $\omega$

After analyzing the influence of cell wall viscoelasticity and intercellular fluid transport (see Sect. 3.3), the next series of simulations focuses on the combined influence of V+FT on the cell wall stretch for different $\omega$. The hypothesis is that the combined influence of the two phenomena (V+FT) and the angular velocity $\omega$ could affect the rate at which cell wall stretch evolves during a stimulus.

We modeled sensory hair deflection up to an angular displacement of $\theta ={0.15}\,\hbox {rad}$ for three different loading rates defined by the angular velocities $\omega =$ ${0.1}\,\hbox {rad s}^{-1}, {0.5}\,\hbox {rad s}^{-1}$, and ${1}\,\hbox {rad s}^{-1}$. Thereafter, we calculated the cumulative stretch $\delta$ at multiple intervals during the stimulus using Eqn. 2 and Eqn. 3 (see Sect. 2.3). The net stretch $\varDelta \delta$ is then found as the difference between $\delta$ at any stage of the deflection and the value of $\delta$ at the beginning. Following this, the variation in $\varDelta \delta$ *vs.* $\theta$ is plotted in Fig. 7a for the sensory cell located at $\phi ={0}\,\hbox {rad}$, which is under maximum tension due to the bending of the hair. When the sensory hair is at its maximum deflection, denoted by $\theta ={0.15}\,\hbox {rad}$, it can be observed that the value of $\varDelta \delta$ is highest (black, $6.7\times 10^{-3}$) for $\omega = 1\,\hbox {rad s}^{1}$, while the lowest value (blue, $4.9\times 10^{-3}$) is obtained for $\omega = {0.1}\,\hbox {rad s}^{-1}$. Furthermore, the stretch rate $\psi _{\omega }$ for the three curves is calculated from the slope of the $\varDelta \delta$ *vs.* $\theta$ curve in the deflection domain ${0.06}\,\hbox {rad}\le \theta \le {0.12}\,\hbox {rad}$ (green shaded). Figure 7b shows the trend of $\psi _{\omega }$ for the three values of $\omega$, which can be approximated using a linear fit having a positive slope of ${0.0203}\,\hbox {s rad}^{-2}$.

## Discussion

The Venus flytrap’s touch-sensitive hairs contain stretch-activated ion channels that are localized in the plasma membrane of the sensory cells (Procko et al. 2021). The gene encoding the MscS-like (MSL) homolog FLYCATCHER1 (FLYC1) was recently reported to show very high expression in sensory cells. The activation kinetics of similar MSLs have been described earlier in the model species *Arabidopsis thaliana* (Haswell and Meyerowitz 2006; Haswell et al. 2008). A total of 10 MSL homologs were identified in *Arabidopsis* and two homologs, namely the MSL9 and MSL10, were observed to be localized in the plasma membrane. In the past, the single-channel patch-clamp technique was used to stimulate MSL10 channels of *Arabidopsis* cells (Maksaev and Haswell 2012). These investigations revealed that the threshold patch-clamp pressure required to open MSL10 channels varies with the applied pressure ramp. This is indicative of a stretch rate-dependent activation of MSLs, and a similar working principle may also be behind the activation of FLYC1.

Stimulus rate-dependency of the flytrap’s response was also evident during our force-deflection tests (Burri et al. 2020), which measured APs only during the sensory hair’s motion, while sustained deflections at maximum angular displacement $\theta$ and deflections with sufficiently low angular velocities $\omega <{0.01}\,\hbox {rad s}^{-1}$ produced no APs. To uncover the mechanistic origins of such a stimulus rate-dependent behavior, a multi-scale FEM hair model was developed to mimic the force-deflection tests at different $\omega$. This is a first of its kind model for the Venus flytrap’s sensory hair, which incorporates both time-dependent material behavior as well as intercellular fluid transport. This way, the model could successfully capture the complex combined influence of cell wall viscoelasticity, fluid transport, and angular velocity $\omega$ in order to examine their individual and combined influences on cell wall stretch. Moreover, the multi-scale arrangement of tissues and cells in our model made it possible to observe the effects of the stimulus specifically on sensory cells, which was otherwise not possible directly *via* experimental characterizations.

The deflection simulations of the advance and retreat phases of the sensory hair revealed that intercellular fluid transport (FT) leads to an increase in the recoverable strain energy SE stored in the cell walls for both elastic (E) and viscoelastic (V) cell walls (see Fig. 5c). This can be explained by the fact that, when a stimulus-generated pressure gradient drives fluid into the cell ($\phi ={0}\,\hbox {rad}$), the incoming fluid further deforms the cell. Meanwhile, the cell at $\phi ={\pi }\,\hbox {rad}$ undergoes compression and, therefore, witnesses an efflux of fluid out of its lumen and into the adjacent cells. The compression causes contraction in the cell walls, which results in negative values for the net cumulative stretch $\varDelta \delta$. At the same time, the effect of viscoelastic cell walls is also highlighted by these simulations, wherein at the maximum angular displacement of $\theta ={0.15}\,\hbox {rad}$, the SE for the two cases V and V+FT is lower than for their elastic counterparts E and E+FT. Interestingly, both V+FT and E+FT cases have SE > 0 at the end of the hair’s retreat since not all of the additional fluid, which had initially entered the cell, has moved out. The effect of FT is more pronounced on $\varDelta \delta$, which witnesses nearly a 72% and 64% increase with respect to the cases of elastic and viscoelastic cell wall behavior. This can be attributed to the competition between the rate of fluid transport $V^{*}$ and the angular velocity $\omega$. Since $V^{*}$ is determined by the membrane’s hydraulic conductivity $L_{p}$, this parameter may indeed be suitably tuned in the flytrap, such that cell deformation also persists during the hair’s retreat, thereby prolonging the deactivation of MSLs or other MSCs. However, the effect of difference in $\varDelta \delta$ for E+FT and V+FT is only 3%, which indicates that fluid transport has a significantly greater effect on $\varDelta \delta$ compared to viscoelasticity.

In the next step, hair deflection tests were simulated with the V+FT combination to derive the resulting sensory cell wall stretch for different $\omega$. We observed that at the maximum deflection, the net cumulative stretch $\varDelta \delta$ increases with increasing values of $\omega$ (see Fig. 7a). This is due to the fact that at higher stimuli rates, the influence of viscoelasticity is less pronounced and, therefore, the hair’s tissues tend to stretch nearly instantaneously, similar to a linear elastic material. This way, our model demonstrates how the macro-scale stimulus parameter $\omega$ exerts an influence at the cellular level, whereby the cell wall is stretched to different extents for the same amount of angular displacement $\theta$. Another important observation is that, at the initial phase of the deflection at $\theta ={0.05}\,\hbox {rad}$, the value of $\varDelta \delta$ is the highest for the slowest stimulus loading rate of $\omega ={0.1}\,\hbox {rad s}$ (blue). This is again an effect of the slow stimulus rate, which allows sufficient time for fluid to flow into the cell, which then amplifies cell wall deformation.

In the final step of this study, we traced the evolution of $\varDelta \delta$ during the stimulus and compared the stretch rates $\psi _{\omega }$ obtained for different values of $\omega$. Here, the variation of $\psi _{\omega }$ *vs.* $\omega$ is approximated using a linear plot. The positive linear slope of $0.0203\,\hbox {s rad}^{-2}$ implies that with increasing $\omega$, not only does the magnitude of cell wall stretch change, but also that the stretch rate increases. However, we remark that this increase is not strikingly rapid, although $\omega$ is varied by an order of magnitude. This may arise from the fact that the stretch is evaluated on the cell wall, while the plasma membrane is not included in this model. Since the physical links between the plasma membrane and the cell wall are reported to be anchor-like structures called Hechtian strands (Liu et al. 2015; Oparka 1994), it is likely that the relaxation behavior of the membrane differs from that of the cell wall itself. Future effort in modeling could be directed at integrating a plasma membrane layer into the sensory cells and evaluate the influence of $\omega$ on the membrane stretch. This could, in-turn, aid in estimating the threshold stretch and stretch rates that may activate MSCs located in the sensory cell’s plasma membrane. In parallel, electrophysiological studies may be attempted on the sensory cells to experimentally quantify activation thresholds, which would then allow for a one-to-one correspondence with the stimulus parameters. In a broader context, these findings on how a stimulus actuates flytraps can be a source of inspiration for bio-inspired sensor applications and actuation of bio-inspired adaptive structures. An exemplary biomimetic system is the Flectofold, a smart shading system inspired by the trap closure of *Aldrovanda vesiculosa*, which can be activated by pneumatic pistons (Körner et al. 2018). While in the case of Flectofold the focus was on the structural design and the actuation mechanism, an application of sensory-hair-like mechanosensors would be a further step toward bio-inspired adaptive and autonomous systems.

## Declarations

### Availability of data and material

The $\mu -$CT data that support the findings of this study are publicly available in 'Micro-CT scans of the Venus flytrap tactile hairs' with the identifier: https://doi.org/10.3929/ethz-b-000460459.
