# Correction to: Mechanical factors contributing to the Venus flytrap’s rate-dependent response to stimuli

**DOI:** 10.1007/s10237-021-01522-9

**Published:** 2021-10-21

**Authors:** Eashan Saikia, Nino F. Läubli, Hannes Vogler, Markus Rüggeberg, Hans J. Herrmann, Ingo Burgert, Jan T. Burri, Bradley J. Nelson, Ueli Grossniklaus, Falk K. Wittel

**Affiliations:** 1 Department of Civil, Environmental and Geomatic Engineering, ETH Zurich, 8093 Zurich, Switzerland; 2 Department of Mechanical and Process Engineering, ETH Zurich, 8092 Zurich, Switzerland; 3 Department of Plant and Microbial Biology and Zurich‑Basel Plant Science Center, University of Zurich, 8008 Zurich, Switzerland; 4 Institut Für Holztechnologie, 01217 Dresden, Germany; 5 Laboratoire de Physique et Mécanique des Milieux Hétérogènes, École Supérieur de Physique et de Chimie Industrielles de la Ville de Paris, 75005 Paris, France; 6 Swiss Federal Laboratories for Material Science and Technology-EMPA, Cellulose and Wood Materials Laboratory, 8600 Dubendorf, Switzerland; 7 Department of Chemical Engineering and Biotechnology, University of Cambridge, Cambridge, CB3 0AS UK

## Correction to: Biomechanics and Modeling in Mechanobiology https://doi.org/10.1007/s10237-021-01507-8

In the original publication of the article, the affiliation “Department of Mechanical and Process Engineering, ETH Zurich, Zurich 8092, Switzerland” was missed for the author “Nino F Läubli”.

The author is affiliated with both “Department of Mechanical and Process Engineering, ETH Zurich, Zurich 8092, Switzerland” and “Department of Chemical Engineering and Biotechnology, University of Cambridge, Cambridge CB3 0AS, United Kingdom”.

The author names in the citations were incorrectly placed outside the brackets. And in several instances the author names of the citation were repeated.

In the eighth reference, the author name was incorrectly given as “Forterre Yoë”, and it should be “Forterre Yoël”.

The original article has been corrected.

